# Stochastic Resonance Reduces Sway and Gait Variability in Individuals With Unilateral Transtibial Amputation: A Pilot Study

**DOI:** 10.3389/fphys.2020.573700

**Published:** 2020-10-19

**Authors:** Aaron D. Likens, Jenny A. Kent, C. Ian Sloan, Shane R. Wurdeman, Nick Stergiou

**Affiliations:** ^1^Center for Research in Human Movement Variability, University of Nebraska at Omaha, Omaha, NE, United States; ^2^Feinberg School of Medicine, Physical Medicine and Rehabilitation, Northwestern University Prosthetics-Orthotics Center, Chicago, IL, United States; ^3^Department of Clinical and Scientific Affairs, Hanger Clinic, Austin, TX, United States; ^4^Department of Environmental, Agricultural, and Occupational Health, College of Public Health, University of Nebraska Medical Center, Omaha, NE, United States

**Keywords:** stochastic resonance, transtibial amputation, pink noise, fractal, rehabilitation

## Abstract

Sub-threshold (imperceptible) vibration, applied to parts of the body, impacts how people move and perceive our world. Could this idea help someone who has lost part of their limb? Sub-threshold vibration was applied to the thigh of the affected limb of 20 people with unilateral transtibial amputation. Vibration conditions tested included two noise structures: pink and white. Center of pressure (COP) excursion (range and root-mean-square displacements) during quiet standing, and speed and spatial stride measures (mean and standard deviations of step length and width) during walking were assessed. Pink noise vibration decreased COP displacements in standing, and white noise vibration decreased sound limb step length standard deviation in walking. Sub-threshold vibration positively impacted aspects of both posture and gait; however, different noise structures had different effects. The current study represents foundational work in understanding the potential benefits of incorporating stochastic resonance as an intervention for individuals with amputation.

## Introduction

The loss of a lower limb introduces mobility challenges and can severely diminish quality of life (Legro et al., 1998; Sinha et al., 2011). In many cases, these challenges can be attenuated by the use of prosthetic devices (Wurdeman et al., 2018), and prostheses are considered essential for restoring routine activities for many individuals with amputation (Esquenazi, 2004). However, despite the advances in prosthetic rehabilitation, limitations persist.

Lower limb amputation is accompanied by the loss of sensory pathways important for proprioception and for the perception of the environment (Antfolk et al., 2013), and, when using a prosthesis, the residual limb is required to perform novel sensory roles (Geurts et al., 1992; Quai et al., 2005). In the absence of tactile sensation and proprioception from the lower extremity (Geurts et al., 1992), prosthesis users can utilize changes in the pressure distribution at the socket-residual limb interface to obtain information with respect to loading and limb orientation (Murphy, 1984). However, the higher tactile acuity of the plantar foot is unmatched in the rest of the limb (Mancini et al., 2014), and pertinent external stimuli may be further attenuated by gel liners and socks worn over the residual limb to aid socket fit and comfort. As compromised somatosensation, both induced experimentally and observed in pathological populations, has been linked to poorer balance and falls (Meyer et al., 2004; Quai et al., 2005; McKeon et al., 2010; Höhne et al., 2012), interventions that target this deficit are warranted.

Although current prosthetic designs may adequately replace the mechanical structures of the amputated limb (Fiedler et al., 2014), there is currently no adequate, non-invasive solution for the sensory loss. In the present study, a novel approach to minimizing the sensory deficits of amputation was explored by aiming to enhance gait- and posture-relevant sensory information conferred by the prosthetic leg.

**Figure 1:**
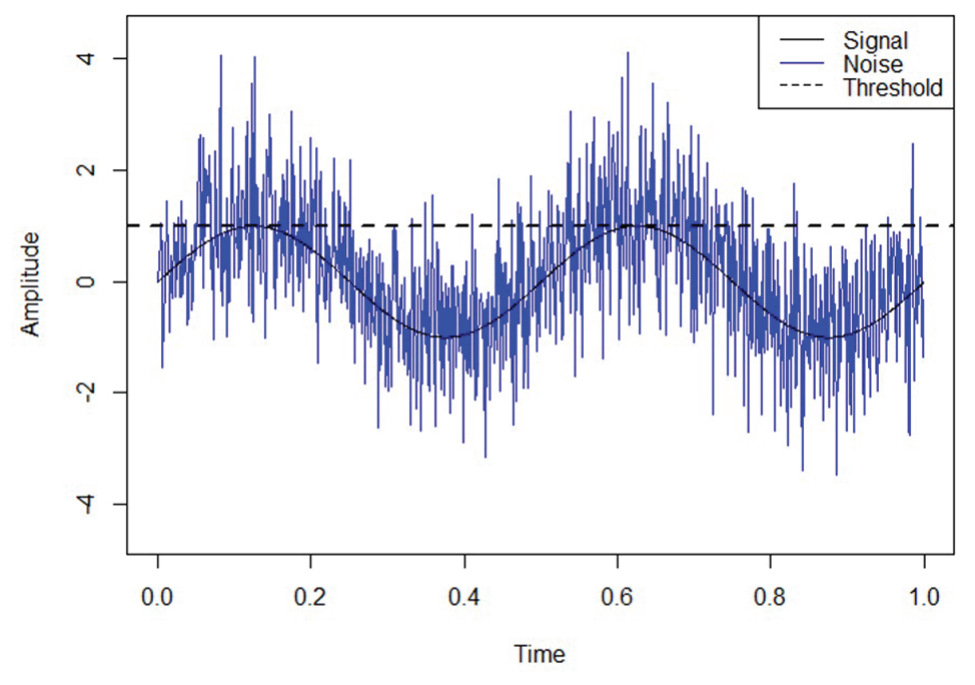
The stochastic resonance concept. The addition of white noise has the effect of providing information about the underlying, sub-threshold signal. In this theoretical example, superimposed noise elevates the signal above threshold, revealing information about its inherent sinusoidal form.

*Stochastic resonance* refers to the phenomenon whereby the presence of noise enhances the perception of weak stimuli or the information content of a signal. This phenomenon has been widely studied in diverse fields such as biology, bioengineering, physics, psychology, and neuroscience (Gammaitoni et al., 1998; Moss et al., 2004; McDonnell and Abbott, 2009). The basic concept of stochastic resonance is captured in Figure 1, depicting how the superposition of noise and signal may enhance the detection of sub-threshold perceptual information. This concept has fueled decades of research involving auditory, haptic, and visual perception (Lugo et al., 2008; Kurita et al., 2013; Krauss et al., 2016; Itzcovich et al., 2017; Pacchierotti et al., 2017). For example, the addition of noise reduces reaction times compared to baseline when applied as transcranial electrical stimulation of the motor cortex (Jooss et al., 2019) and also enhances action-observation task performance and improves three-dimensional perception (Ditzinger et al., 2000; Sasaki et al., 2006). The application of stochastic noise to the wrist increases detection rates of delayed visual stimuli (Nobusako et al., 2018). Integration of stochastic noise decreases sensory thresholds and increases signal detection rates (Richardson et al., 1998; Wells et al., 2005; Iliopoulos et al., 2013; Kurita et al., 2013; Sueda et al., 2013). Hence, the stochastic resonance phenomenon implies that the presence of noise generally enhances neural and perceptual processes. A subsequent implication is that stochastic resonance may be leveraged in applied settings (Moss et al., 2004).

The stochastic resonance phenomenon has been applied in a number of cases involving sensorimotor dysfunction. The guiding hypothesis behind this approach is that by applying sub-threshold vibration to the skin superficial to damaged nerves, the weak afferent signals are enhanced (Moss et al., 2004). For example, application of sub-threshold vibration to the plantar surface of the foot reduces sensory thresholds at the extremities of patients with neuropathy (Liu et al., 2002; Khaodhiar et al., 2003; Zwaferink et al., 2018) and decreases step width while increasing step length in older adult fallers (Galica et al., 2009; Stephen et al., 2012). Furthermore, noise-based techniques have been shown to decrease sensory thresholds and improve balance control in older adults, healthy young adults, and stroke survivors (Gravelle et al., 2002; Priplata et al., 2003, 2006; Costa et al., 2007; Magalhães and Kohn, 2011; Toosizadeh et al., 2018). Collectively, these studies provide empirical support for the stochastic resonance hypothesis while providing strong empirical support for application in therapeutic settings.

**Figure 2:**
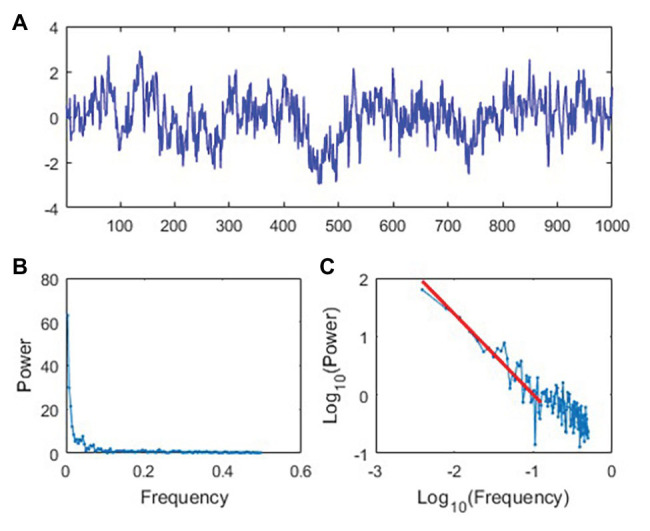
Demonstration of pink noise structure. **(A)** Simulated time series data representing 1/f^β^ with a pink noise structure. The vertical axis has arbitrary units. **(B)** Power spectral density for the time series in **(A)** represented on linear axes. **(C)** Power spectral density for the time series in **(A)** represented on base-10 logarithmic axes.

Stochastic resonance research in rehabilitation may benefit from a number of findings demonstrating that noise expressed by human physiology and behavior rarely, if ever, is simple noise (Goldberger et al., 2002; Kello et al., 2010; Diniz et al., 2011; Harrison and Stergiou, 2015; Cavanaugh et al., 2017). Numerous physiological and behavioral researchers have documented time series patterns which have been termed 1/*f^β^* noise (Gilden et al., 1995; Gilden, 2001; Goldberger et al., 2002; Lipsitz, 2002; Jordan et al., 2007a,b; Kello et al., 2010; Diniz et al., 2011; Ihlen and Vereijken, 2013; Hunt et al., 2014; Likens et al., 2014, 2015; Cavanaugh et al., 2017; Vaz et al., 2019). The term 1/*f^β^* noise originates from the observation that, when decomposed by a discrete Fourier transformation, these time series have frequency spectra within which the amplitude of a given component varies inversely with its frequency (Figures 2B,C). That is, the relation between frequency (f) and amplitude (A) for such noise processes are described by the formula A = 1/*f^β^*, where the exponent *β* is the slope of the linear relationship between log A and log f. This power law behavior in the frequency domain is directly related to fractal properties (e.g., self-similarity) in time series data (Eke et al., 2000; Stroe-Kunold et al., 2009) that have been well documented in healthy forms of human movement (Hausdorff et al., 1995; Hausdorff, 2007). For example, the exponent *β* is related to the so-called Hurst exponent, *H*, commonly used in the study of fractals by *H* = (*β* + 1)/2 in the case of stationary time series and *H* = (*β* − 1)/2 in the case of nonstationary time series. The behavior of a 1/*f^β^* noise signal depends on the value of *β* observed. For example, *β* = 0 denotes a white noise process that exhibits no serial dependence (i.e., no autocorrelation). When *β* = (0, 1), the series represents a pink noise process with positive autocorrelation that decays slowly over time. Lastly, blue noise is present when *β* = (−1, 0) and is characterized by negative autocorrelation that decays rapidly over time. Pink noise (e.g., Figure 2A) is of particular relevance for behavioral and physiological measurements that exhibit serial dependence over timescales representing seconds, minutes, hours, and even days (Stergiou et al., 2016).

In part, the importance of 1/*f^β^* phenomena is apparent from its general pervasiveness (Kello et al., 2008). More important, though, is the observation that classes of 1/*f^β^* noise, such as pink noise and white noise, are commonly observed in cases of health and disease, respectively (Hausdorff et al., 1997; Ivanov et al., 2001; Goldberger et al., 2002; Stergiou et al., 2006; Diniz et al., 2011; Van Orden et al., 2011; Kaipust et al., 2012, 2013). This prevalent relationship between *β* and health has led researchers to hypothesize that external sources of 1/*f^β^* noise might stimulate patterns of variation similar to those observed in cases of health, aging, and disease (Baram and Miller, 2007; Hove et al., 2012; Sejdić et al., 2012; Hunt et al., 2014). For example, when people synchronize their gait with 1/*f^β^* noise similar to pink or white noise, their gait structure resembles those forms (Hunt et al., 2014). Moreover, center of pressure (COP) during quiet standing in older adults exhibits the characteristics of a 1/*f^β^* time series (Manor et al., 2010).

Based on the stochastic resonance paradigm, applying sub-threshold vibration to the affected limb of a person with amputation may be a means to augment the weak afferent signals from the residual limb (Chen et al., 2016; Wan et al., 2016; Crea et al., 2017). It is plausible that the use of vibration with a more biologically-relevant 1/*f^β^* structure may provide similar or enhanced benefit. This hypothesis is supported by numerous modeling and *in vitro* animal studies demonstrating enhanced signal detection in neural circuits in the presence of pink noise as compared to white noise (Nozaki and Yamamoto, 1998; Nozaki et al., 1999a,b; Duan et al., 2014; Qu et al., 2019; Castellanos and Kogelbauer, 2020). The current work takes an initial step in that direction, by comparing effects of white and pink noise vibration on key parameters of gait and postural control.

**Figure 3:**
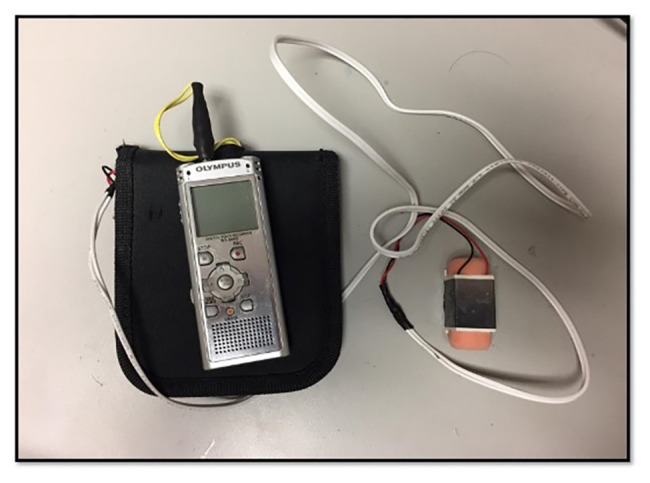
Vibration apparatus. The audio player and amplifier (within black case) were stored in a backpack, and the tactor secured to the affected limb of the participant. Cabling was restrained using Coban wrap (3M, St. Paul, MN, United States) to prevent catching, ensuring there was no restriction to movement.

The purpose of this work was to determine whether the application of sub-threshold vibration could improve standing balance and gait in people with unilateral transtibial amputation. For this purpose, we applied sub-threshold mechanical vibration to the affected limb of transtibial prosthesis users using a lightweight tactor (Figure 3). We assessed the effect of noise signals with pink and white frequency spectra, in comparison to no vibration, on postural sway during quiet standing and on speed and stride characteristics during walking. It was hypothesized that, when applied to the affected limb, sub-threshold vibration would improve various aspects of gait and posture due to the enhancement of sensory information. It was further hypothesized that these improvements would be most prominent for stimuli that most closely approximated healthy 1/*f^β^* noise patterns (i.e., pink noise).

## Materials and Methods

### Study Design

All procedures were approved by the University of Nebraska Medical Center Institutional Review Board and the VA Nebraska-Western Iowa Health Care System Institutional Review Board. All tests were conducted within a university motion capture laboratory. Twenty-one people with unilateral transtibial amputation (Table 1) were recruited from prosthetic clinics around the Omaha/Metropolitan area and local VA hospital. Participants were screened prior to testing and excluded based on the following criteria: amputation less than 6 months ago, any neurological disease/impairment that may affect gait (except diabetes), currently pregnant, and/or any sores on the residual limb. All participants provided written, informed consent prior to the start of testing. Standing and walking were assessed in the first and second of two sessions, respectively.

**Table 1:** Participant information.

Cause	Age (years)	Height (m)	Mass (kg)	Years since amp.	Gender
Higher threshold group (*n* = 11; eight completed walking trials)					
Trauma (*n* = 3)	53.3	1.83	112.3	7.3	3M
Diabetes (*n* = 2)	59.0	1.79	91.2	3.5	2M
Infection (*n* = 3)	66.6	1.81	113.9	4.0	2M, 1F
Other (*n* = 3)	69.6	1.81	92.8	16.2	3M
All (*n* = 11)	62.1 ± 6.4	1.81 ± 0.01	102.6 ± 10.6	7.8 ± 5.1	(10M, 1F)
Lower threshold group (*n* = 9; eight completed walking trials)					
Trauma (*n* = 5)	51.00	1.78	96.52	7.66	3M, 3F
Diabetes (*n* = 2)	67.00	1.81	104.10	10.25	2M
Infection (*n* = 0)	-	-	-	-	-
Other (*n* = 2)	51.50	1.73	98.66	20.15	1M, 1F
All	56.50 ± 7.42	1.77 ± 0.03	99.76 ± 3.19	12.69 ± 5.38	(6M, 4F)

Average values for higher and lower light touch threshold groups (mean ± standard deviation), separated by cause of amputation.

### Experimental Design

#### Intervention

Vibration was produced using a BM3C tactor (TactileLabs, Montreal, Canada) connected to an audio player (Olympus, Center Valley, PA, United States) *via* an in-house built amplifier. Twenty-minute pink and white noise signals were created using version 2.3.2 of Audacity®, and an envelope filter was applied to account for the non-linearity in tactor output.

All tests were conducted under three vibration conditions: none, pink, and white. Participants were blinded to condition, and order was randomized across participants.

#### Procedures

Vibration amplitudes for the pink and white noise signals were determined independently at the start of each session, with signal order randomized. The tactor (Figure 3) was attached anteriorly to the mid-thigh of the affected limb using hypoallergenic double-sided tape and secured with Cover-Roll stretch tape (BSN Medical, Charlotte, NC, United States). The distance from the proximal border of the patella to the middle of the device was measured to maintain consistency across sessions. The audio player and amplifier were stored in a backpack worn by the participant.

The participant was asked to stand with support from a walking frame, to wear headphones to block all other noise during the testing, and to fixate on a wall mounted cross. A 4-2-1 method (Dyck et al., 1993) was used to determine the threshold of perception for each signal, with adjustments made using the discrete volume levels of the audio player. The participant’s ability to feel vibration was signaled by hand gesture, with fist-open and fist-closed postures indicating sensation and lack of sensation, respectively. Following threshold determination, the amplitude was reduced by 1–2 levels, such that the applied vibration was between 60 and 90% of the threshold of perception of the participant. For each signal, the amplitude was evaluated and confirmed to be within this range using an oscilloscope (DS1102E Rigol Technologies Inc. Beaverton, OR, United States). The discrete increments of the audio player prevented further precision; however, values spanning this threshold range have been successfully utilized in previous studies utilizing the stochastic resonance paradigm (Collins et al., 2003; Priplata et al., 2003, 2006; Enders et al., 2013).

#### Data Collection

Participants completed a 90-s quiet standing trial in each vibration condition. Participants were asked to stand on dual floor-embedded force plates (Optima, AMTI, Watertown) with their feet as close together as possible and to cross their arms across their chest, to induce a moderate challenge to balance. They were asked to look directly at a cross placed at eye level on the wall in front of them. Force plate data were recorded for 90 s at 600 Hz in each vibration condition.

Walking tests were conducted within the central measurement space of the motion laboratory. Participants performed at least 10 traverses in each vibration condition, at a self-selected pace. Kinematic data from reflective markers placed on the pelvis and feet were collected at 100 Hz with a 12-camera motion capture system (Motion Analysis Corporation, Santa Rosa, CA, United States). A 5-min rest period was enforced between vibration conditions.

### Data Processing

Kinematic data for walking trials were tracked using Cortex (Motion Analysis Corporation, Santa Rosa, CA, United States). All further processing was performed in Visual 3D (C-Motion, Germantown, MD, United States). Fourth order low pass Butterworth filters with a 6 Hz cutoff for kinetic standing data and a 7 Hz cutoff for kinematic walking data were applied, determined using the methods of residuals as described by Winter (2009).

**Figure 4:**
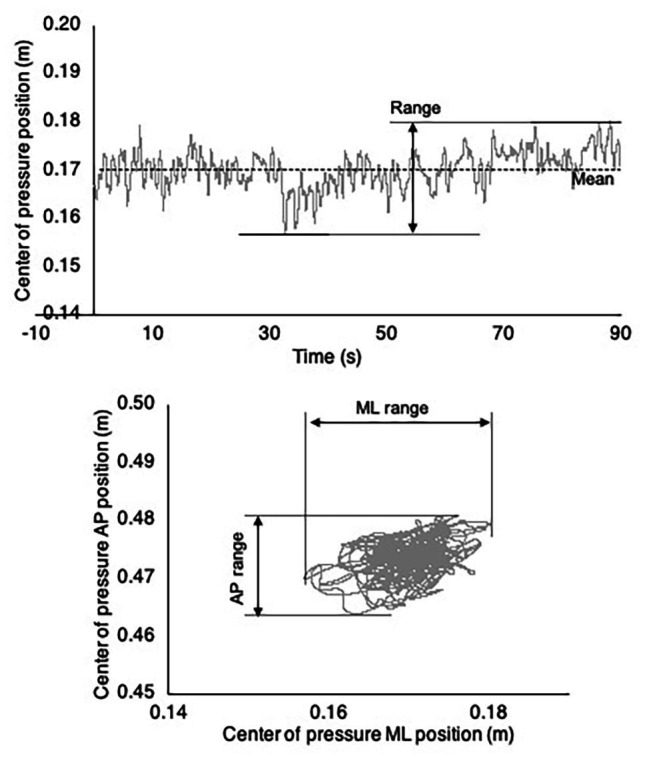
Example center of pressure (COP) time series and trajectory. (Top) Example mediolateral COP time series. The COP range is labeled and indicated by horizontal lines. (Bottom) Complete COP trajectory for the example COP time series depicted in the top panel.

The center of pressure (COP) during quiet standing was calculated from the combined output of the two force plates. The range and root-mean-square (RMS) displacement of the COP were computed in the mediolateral and anterior-posterior directions across each 90-s trial. An example COP time series is depicted in Figure 4.

Bilateral foot contact events during walking were algorithmically estimated based on marker positions, utilizing the relative velocities of the feet and pelvis (Zeni et al., 2008). Step lengths were calculated as the distance in the direction of travel between the ankle joint centers at contralateral and ipsilateral foot contacts. Step width was calculated as the distance between the ankle joint centers in the direction perpendicular to the direction of travel. For each vibration condition, step lengths and step widths from 10 strides on each limb (prosthetic and sound) were extracted and the average (mean) and variability (standard deviation) were computed. Additionally, average walking speed was calculated for each walking traverse.

### Statistical Analysis

Participants were grouped retrospectively into lower and higher light touch perception threshold groups according to their ability to detect a 10-g Semmes-Weinstein monofilament applied to the tibial crest of their residual limb. Although not a primary aim of this study, our rationale when dividing the participants into these two sensory groups was to explore whether individuals who originally exhibited higher sensory thresholds (less sensitive) might benefit more or less than those who exhibited lower sensory thresholds. To address this and the main hypotheses of the study, a series of linear mixed effects models were constructed to understand the effects of vibration condition and sensory threshold on the measures presented in the previous section. Linear mixed effects (LME) models (aka multilevel models and hierarchical linear models) are contemporary alternatives to standard regression and analysis of variance frameworks that do not carry the same unrealistic assumptions (Raudenbush and Bryk, 2002). For example, whereas the standard regression framework enforces a common intercept, the LME framework allows the user to estimate unique intercepts for each participant explicitly. Estimation of random slopes is also possible but not used in this paper. Modeling procedures for this approach entail starting with a baseline model and adding terms sequentially to form a series of nested models. Subsequent models are tested for improvement in fit *via* likelihood ratio tests involving *χ*^2^ statistics. In the present case, we defined our baseline models as “intercept-only” models, that is, models that account for average behavior of each participant in our study. Such intercept-only models are generally not interpreted but are used as a point of comparison instead by which to gauge relative importance of focal predictors (i.e., fixed effects). Our modeling strategy involved two steps: (1) Intercept-only models were fit for each dependent measure. (2) A second model was constructed for each dependent measure that contained fixed main effects and interactions of *noise type* and *light touch threshold group* (higher and lower), in addition to random intercepts from (1). Fixed effects and interactions were added in a single modeling step as *a priori* predictions about precedence between condition and threshold variables were not made. Log-likelihood tests were used to assess improvements in the model fit that result from the combination of noise type and light touch threshold group.

## Results

Of 21 participants recruited for the study, 20 participants completed standing tests only and 16 completed standing and walking tests. One participant was withdrawn completely due to dizziness. One participant did not attend the second session due to an injury that required surgery. Two further declined to participate, and data from one participant were excluded from walking analysis due to use of a powered ankle. Eleven participants were placed in a higher threshold group and nine in a lower threshold group (Table 1) based on their ability to perceive a 10-g monofilament applied at the tibial crest of their affected limb. In the following results, we only report those models that were statistically significant, namely, COP range and RMS during standing, and sound limb step length variability during walking. Complete data for these measures and demographics may be found in supplementary material.

### Postural Control During Quiet Standing

**Figure 5:**
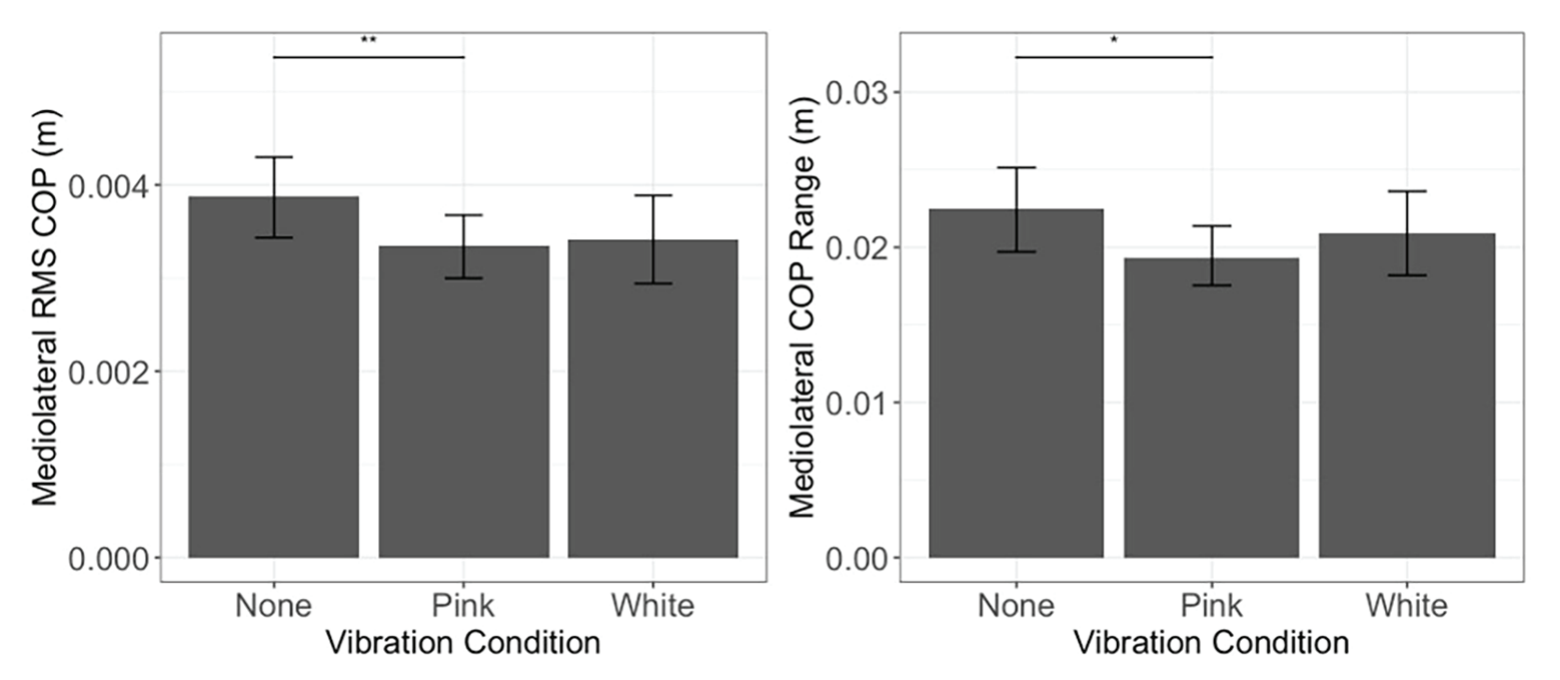
Postural control during quiet standing. Mediolateral COP root-mean-square (RMS; left) and range (right) during quiet standing as a function of vibration condition. Error bars reflect 95% confidence intervals. Range and RMS were height normalized. ^**^p < 0.01 and ^*^p < 0.05. No differences were observed between light touch threshold groups, and no interactions were observed with respect to these groups. Hence, means and confidence intervals depicted here collapse across light touch threshold.

Linear mixed effect models were used to assess whether inclusion of fixed effects of noise and threshold group, along with their interactions, improved prediction of COP excursion over and above an intercept only model. In the mediolateral COP range, the addition of fixed effects and interactions improved the model fit [*χ*^2^(5) = 11.06, *p* = 0.05]. Improvement in the model fit stemmed from the fact that participants exhibited reduced mediolateral COP range in the pink condition when compared to the no noise (none) condition (estimate = −0.004, *SE* = 0.002, *p* = 0.013; Figure 5, right). Moreover, the addition of fixed effects and interactions improved the model for COP RMS [*χ*^2^(5) = 17.81, *p* = 0.003], showing a reduction in COP RMS in the pink noise condition when compared to the no noise (none) condition (estimate = −0.001, *SE* = 0.0002, *p* = 0.008; Figure 5, left). Our analysis revealed no further significant differences for sway during quiet standing. Model building steps and tests for models in Postural Control During Quiet Standing and Speed and Stride Characteristics During Walking sections are summarized in Table 2.

**Table 2:** Summary of model building steps for linear mixed effects models presented in sections Postural Control During Quiet Standing and Speed and Stride Characteristics During Walking.

Model	AIC	BIC	Log likelihood	Deviance	*χ*^2^	*DF*	*p*
Sound limb step length variability							
Baseline model	−336.00	−331.01	171.00	−342			
Baseline + Noise × Group	−341.69	−328.38	178.84	−357.69	15.69	5	0.008
Mediolateral COP range							
Baseline model	−307.43	−302.44	156.72	−313.43			
Baseline + Noise × Group	−308.49	−295.18	162.25	−324.49	11.06	5	0.050
Mediolateral COP RMS							
Baseline model	−451.13	−446.14	228.56	−457.13			
Baseline + Noise × Group	−458.94	−445.63	237.47	−474.94	17.81	5	0.003

### Speed and Stride Characteristics During Walking

**Figure 6:**
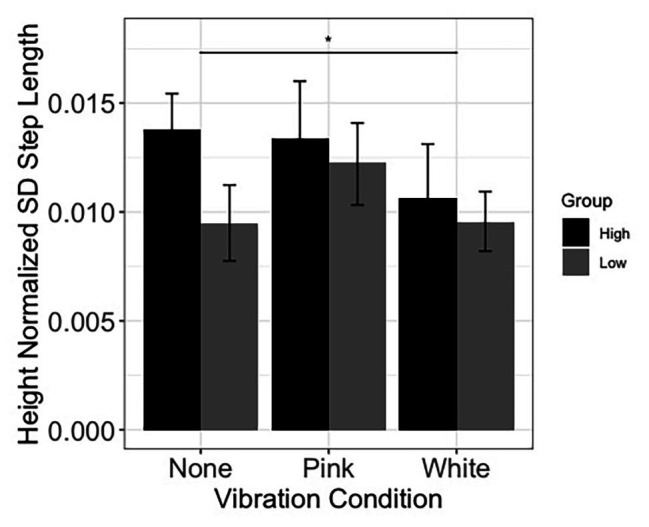
Sound limb step length variability during walking. Results displayed as a function of vibration condition and light touch threshold group (higher and lower denote individuals with higher and lower thresholds of perception to light touch, measured at the tibial crest). Error bars reflect 95% confidence intervals. SD, standard deviation. The horizontal bar reflects the comparison between the none and white noise conditions.

Linear mixed effect models were used to assess whether inclusion of fixed effects of noise and threshold group, along with their interactions, improved prediction of speed and stride characteristics over and above an intercept only model. Models involving speed and step length were not improved by addition of fixed effects and interactions. Concerning sound limb step length variability, the addition of fixed effects and interactions improved the model fit [*χ*^2^(5) = 15.69, *p* = 0.008]. The analysis further revealed a decrease in sound limb step length variability in the white noise condition compared to the no noise (none) condition (estimate = −0.003, *SE* = 0.001, *p* = 0.034; see Figure 6). Also, participants who were in the higher light touch threshold (less sensitive) group produced a greater sound limb step length variability than participants in the lower threshold group (estimate = −0.004, *SE* = 0.002, *p* = 0.008; see Figure 6). No other gait comparisons were significant.

## Discussion

In this study, two primary hypotheses were explored related to the use of sub-threshold vibration to augment afferent information of the affected limbs of people with amputation: (1) application of sub-threshold vibration would improve various aspects of standing and walking and (2) improvements would be greatest for stimuli that more closely approximated healthy forms of 1/*f^β^* noise. In brief, results provide promising, albeit limited, evidence in favor of both hypotheses. Specifically, white noise stimulation decreased sound limb step length variability during walking when compared to no stimulation. With respect to the second hypothesis, mediolateral sway in the standing trials decreased during the pink noise stimulation when compared to the no stimulation condition.

As noted above, white noise stimulation of the affected limb reduced step length variability of the sound limb. Two issues make this finding challenging to interpret. First, the result seems counterintuitive because no stimulation was applied to the sound limb. It is possible that the control of the affected limb during single limb stance improved, resulting in a reduced variability of foot placement of the sound limb in the subsequent step. Vibration was provided throughout the gait cycle, but effects of stimulation may only be evident during the sound limb swing phase when the prosthetic limb makes prolonged contact with the ground. An alternative explanation is that this effect is similar in nature to what is found in cases of cross education (Hendy and Lamon, 2017). Cross education refers to the phenomenon where unilateral resistance training results in strength gains in an untrained but homologous muscle group. Accordingly, perceptual training of the affected limb with sub-threshold noise could produce consonant perceptual gains in the sound limb. This seems unlikely, however, because the affected limb did not show reliable improvements in the white noise condition and our manipulations did not include prolonged training.

Second, and contrary to our predictions, white noise reduced sound limb step length variability while pink noise did not have any apparent effect. While this result was surprising with respect to the hypotheses, the influence of white noise stimulation on step and stride length variability in older adults has been documented elsewhere (Stephen et al., 2012; Lipsitz et al., 2015). Hence, the present results appear consistent with those findings. Furthermore, the optimal noise pattern may depend on the task. Local non-stationarities present in pink noise imply that white noise reverts to its mean faster than pink noise, which in turn suggests the possibility that white noise more consistently enhances sensory information during irregular walking patterns. In contrast, pink noise may be better for behaviors involving more continuous stimulation such as postural control during standing. Further, we note that, in some instances, adding white noise into perception-action loops may cause alterations in the typical pink noise structure often observed in healthy biological signals (Dotov et al., 2010).

In contrast to the gait findings, the results concerning pink noise and postural sway provided support for both our first and second hypotheses. Specifically, it was noted that pink noise reduced sway in the mediolateral direction when compared with no stimulation. These results are consistent with the idea that augmenting afferent signals with pink noise may be more beneficial for stabilizing posture than other forms of noise. The source of this benefit may relate to the documented patterns of pink noise in behavior (Manor et al., 2010; Hunt et al., 2014) and in the nervous system (Voytek et al., 2015; Cohen, 2016; Harrison et al., 2018). That is, pink noise may be better suited to augment afferent feedback because of its similarity to other naturally occurring physiological noise properties. Confidence in these results, however, is hampered by the seeming contradiction between the results for gait and those for posture. Future research will be needed to resolve this inconsistency.

A potential conclusion is that white noise may be more effective at augmenting gait-related information, while pink noise may better convey posture-relevant information through afferent channels. We draw this conclusion cautiously, because although statistical evidence favors this interpretation, replications will be needed to establish clinical significance. Furthermore, we only observed stochastic resonance effects relative to mediolateral control of postural sway; no effects were observed in the anterior-posterior direction. One possible explanation for limited efficacy may be the lack of volitional control of the prosthetic foot, which may impede the influence of vibratory stimuli on the anterior-posterior control of sway. Further distinguishing between gait and posture related effects will require a broader study of both the magnitude and structure of noise used for augmenting sensory information. Indeed, others have demonstrated means to estimate the optimal magnitude of noise in terms of mutual information between spike trains from threshold crossings and the signal itself – too little noise will have little effect on sensory threshold crossings, too much noise will mask the signal completely (Moss et al., 2004; Wells et al., 2005). Perhaps, a similar approach should be applied for determining the optimal structure of noise based on the intrinsic noise properties of the underlying signal.

### Limitations and Future Directions

The current results hint at a differential effect of sub-threshold vibration on gait, as compared to posture; however, there are several limitations that would need to be resolved to form such a conclusion. One limitation is that we did not measure the relationship between noise amplitude and gait and posture outcomes. While beneficial effects have been previously observed using a similar range of noise amplitudes to those applied here (Liu et al., 2002; Priplata et al., 2003; Enders et al., 2013; Lipsitz et al., 2015), it is possible that noise levels were not optimal for each participant. In a sense, this limitation echoes our comments from the preceding paragraphs and underscores a need for further systematic study of noise characteristics in varying contexts. Another limitation is that differences between the vibration thresholds for walking and standing may have contributed to this pattern of results. The setting for the vibration threshold was determined during standing. It has been shown that vibration threshold significantly increases in a standing posture when compared to a sitting posture (Mildren et al., 2016). Hence, the threshold during walking may fluctuate both below and above that derived during standing as the limb is loaded and offloaded. This could be counteracted by setting independent vibration amplitudes for standing and for different phases of gait (Stephen et al., 2012). For example, appropriate vibration thresholds could be established while the participant is engaged in a gait task, or for a range of poses such as laying down, standing on both legs, and standing with the affected leg raised off the ground, and vibration amplitudes set accordingly.

The placement of the tactor within the present experiment may be a limitation. Although previous studies have demonstrated that perceptual acuity may be enhanced by sub-threshold noise applied proximal to, and not directly at, the site of interest (Breen and Macefield, 2013; Breen et al., 2016), it is possible that placement closer to the site of amputation may have resulted in a greater effect. It is indeed unclear whether the observed results may have been due to an effect on cutaneous or proprioceptive channels, or a combination. Although vibration amplitudes were set individually, it is also possible that differences in body composition may have affected efficacy on an individual level, due to differential effects on the superficially applied vibration. Future research should address these issues more explicitly by testing different placement locations with the aim of uncovering what specific afferent information is enhanced by vibratory stimulation and whether alternative placement sites would optimize stochastic resonance effects. Such work may be challenging, however, given the observation that stochastic resonance effects can be quite general, even crossing sensory modalities (Lugo et al., 2008). In the current study our choice for placement was likewise guided by practical concerns. We observed substantial changes in signal content when the tactor was attached directly to the socket of the prosthesis. The chosen location permitted consistent positioning across participants, without the requirement to alter individual prosthesis suspension methods, e.g., prosthetic sleeves, while still applying stimulation to the affected limb.

To recapitulate, the goal of the current research was to improve the control of gait and posture by enhancing afferent information about the prosthetic limb. Yet, our results indicate that different contexts (e.g., walking vs. standing) promote different sensitivities to categorically different noise types. In addition to the mechanical and physiological considerations already discussed, those results raise questions about the organization of the cognitive-perceptual system and its interactions with the environment. That is, the context-dependence implied in our results situates them within the broader theoretical landscape of complex dynamical systems theory. This theoretical approach suggests that cognitive-perceptual systems self-organize to points of criticality that allow the system to quickly adapt to novel circumstances (Van Orden et al., 2003). The specific organization of a system and its putative sensitivities, then, may depend on the specific set of constraints imposed on the entire cognitive-perceptual system involved in the control of walking and maintaining upright posture. Hence, future work would benefit from a systematic investigation of such context-dependent sensitivities. The present study only explored two categorically different forms of noise: white noise and pink noise. These signals represent prototypical, albeit simplified, patterns of statistical independence and persistence. In reality, these classes rest on a continuum, ranging from strongly antipersistent to strongly persistent. Future work should explore a greater range of these possible structures and directly compare those structures in terms of resistance to habituation.

The current paper relied on linear measures (e.g., means and standard deviations) to make inferences about the effects of stochastic resonance on the control and stability of gait and posture. However, a large literature suggests that, in addition to linear outcome measures, it is critical to examine the temporal (e.g., fractal) properties these behaviors exhibit (Hausdorff et al., 1995; Hausdorff, 2005; West and Latka, 2005; Xue et al., 2016; Phinyomark et al., 2020) that often covary with cases of aging and neurodegenerative disease (Hausdorff, 2007, 2009). While we did not investigate these properties in the present study, we might expect to see similar trends in individuals with lower limb amputation. That is, we anticipate that, relative to healthy controls, people with amputation may show evidence of reduced statistical persistence in gait and posture dynamics, as measured by fractal scaling exponents. Furthermore, we also anticipate that stochastic resonance may attenuate such effects. Further research will be needed to explore those hypotheses.

### Conclusion

Stochastic resonance has figured prominently in the study of physiological and perceptual processes. Many studies investigating stochastic resonance have used a form of white noise as a means to augment sensation. In this paper, the use of pink noise, which is more reflective of healthy biological forms of variability, was proposed with the aim of improving standing and walking performance in individuals with a unilateral transtibial amputation. The current results provide tentative support for this proposition. In particular, it was found that while white noise reduced step length variability during walking, pink noise reduced mediolateral sway during quiet standing. A major challenge for future research replicating these findings will be to discover the neuromechanical mechanisms responsible for any differential effects of noise with respect to control of gait and posture. Hence, while not without limitations, the current results suggest a number of possible areas for future research.

## Data Availability Statement

The data supporting the conclusions of this article have been made available as Supplementary Material.

## Ethics Statement

The studies involving human participants were reviewed and approved by University of Nebraska Medical Center Institutional Review Board and the VA Nebraska-Western Iowa Health Care System Institutional Review Board. The patients/participants provided their written informed consent to participate in this study.

## Author Contributions

AL contributed to writing the original draft, revising and editing the manuscript, statistical analysis, and visualizations. JK contributed to the methodology development, data collection, and revising and editing the manuscript. CS contributed to data collection, writing the original draft, and editing the manuscript. SW contributed to conceptualization of the study, methodology development, and revising and editing the manuscript. NS contributed to conceptualization of the study, methodology development, and revising and editing the manuscript. All authors contributed to the article and approved the submitted version.

### Conflict of Interest

The authors declare that the research was conducted in the absence of any commercial or financial relationships that could be construed as a potential conflict of interest.

## References

[ref1] Antfolk C. D’Alonzo M. Rosén B. Lundborg G. Sebelius F. Cipriani C. (2013). Sensory feedback in upper limb prosthetics. Expert Rev. Med. Devices 10, 45–54. 10.1586/erd.12.68, PMID: 23278223

[ref2] Baram Y. Miller A. (2007). Auditory feedback control for improvement of gait in patients with multiple sclerosis. J. Neurol. Sci. 254, 90–94. 10.1016/j.jns.2007.01.003, PMID: 17316692

[ref3] Breen P. P. Macefield V. G. (2013). “Proximally applied subsensory electrical noise stimulation reduces variance in action potential timing and enhances sensory perception” in *6th International IEEE/EMBS Conference on Neural Engineering (NER);* November 2013; 267–270.

[ref4] Breen P. P. Serrador J. M. O’Tuathail C. Quinlan L. R. McIntosh C. ÓLaighin G. (2016). Peripheral tactile sensory perception of older adults improved using subsensory electrical noise stimulation. Med. Eng. Phys. 38, 822–825. 10.1016/j.medengphy.2016.05.015, PMID: 27317362

[ref5] Castellanos A. P. Kogelbauer F. (2020). Pink noise amplifies stochastic resonance in neural circuits. Eng. Res. Exp. 10.1088/2631-8695/ab8442 [Epub ahead of print]

[ref6] Cavanaugh J. T. Kelty-Stephen D. G. Stergiou N. (2017). Multifractality, interactivity, and the adaptive capacity of the human movement system: a perspective for advancing the conceptual basis of neurologic physical therapy. J. Neurol. Phys. Ther. 41, 245–251. 10.1097/NPT.0000000000000199, PMID: 28834791 PMC5676558

[ref7] Chen B. Feng Y. Wang Q. (2016). Combining vibrotactile feedback with volitional myoelectric control for robotic transtibial prostheses. Front. Neurorobot. 10:8. 10.3389/fnbot.2016.00008, PMID: 27597824 PMC4993021

[ref8] Cohen M. X. (2016). Midfrontal theta tracks action monitoring over multiple interactive time scales. NeuroImage 141, 262–272. 10.1016/j.neuroimage.2016.07.054, PMID: 27475291

[ref9] Collins J. J. Priplata A. A. Gravelle D. C. Niemi J. Harry J. Lipsitz L. A. (2003). Noise-enhanced human sensorimotor function. IEEE Eng. Med. Biol. Mag. 22, 76–83. 10.1109/MEMB.2003.1195700, PMID: 12733463

[ref10] Costa M. Priplata A. A. Lipsitz L. A. Wu Z. Huang N. E. Goldberger A. L.. (2007). Noise and poise: enhancement of postural complexity in the elderly with a stochastic-resonance–based therapy. Europhys. Lett. 77:68008. 10.1209/0295-5075/77/68008, PMID: 17710211 PMC1949396

[ref11] Crea S. Edin B. B. Knaepen K. Meeusen R. Vitiello N. (2017). Time-discrete vibrotactile feedback contributes to improved gait symmetry in patients with lower limb amputations: case series. Phys. Ther. 97, 198–207. 10.2522/ptj.20150441, PMID: 28204796

[ref12] Diniz A. Wijnants M. L. Torre K. Barreiros J. Crato N. Bosman A. M. T.. (2011). Contemporary theories of 1/f noise in motor control. Hum. Mov. Sci. 30, 889–905. 10.1016/j.humov.2010.07.006, PMID: 21196059

[ref13] Ditzinger T. Stadler M. Strüber D. Kelso J. A. S. (2000). Noise improves three-dimensional perception: stochastic resonance and other impacts of noise to the perception of autostereograms. Phys. Rev. E Stat. Phys. Plasmas Fluids Relat. Interdiscip. Topics 62, 2566–2575. 10.1103/physreve.62.2566, PMID: 11088737

[ref101] Dotov D. G. Nie L. Chemero A. (2010). A demonstration of the transition from ready-to-hand to unready-to-hand. PLoS One 5:e9433. 20231883 10.1371/journal.pone.0009433 PMC2834739

[ref14] Duan F. Chapeau-Blondeau F. Abbott D. (2014). Stochastic resonance with colored noise for neural signal detection. PLoS One 9:e91345. 10.1371/journal.pone.0091345, PMID: 24632853 PMC3954722

[ref15] Dyck P. J. O’Brien P. C. Kosanke J. L. Gillen D. A. Karnes J. L. (1993). A 4, 2, and 1 stepping algorithm for quick and accurate estimation of cutaneous sensation threshold. Neurology 43, 1508–1512. 10.1212/wnl.43.8.1508, PMID: 8351003

[ref16] Eke A. Herman P. Bassingthwaighte J. Raymond G. Percival D. Cannon M.. (2000). Physiological time series: distinguishing fractal noises from motions. Pflugers Arch. 439, 403–415. 10.1007/s004249900135, PMID: 10678736

[ref17] Enders L. R. Hur P. Johnson M. J. Seo N. J. (2013). Remote vibrotactile noise improves light touch sensation in stroke survivors’ fingertips via stochastic resonance. J. Neuroeng. Rehabil. 10:105. 10.1186/1743-0003-10-105, PMID: 24112371 PMC3852405

[ref18] Esquenazi A. (2004). Amputation rehabilitation and prosthetic restoration. From surgery to community reintegration. Disabil. Rehabil. 26, 831–836. 10.1080/09638280410001708850, PMID: 15497912

[ref19] Fiedler G. Akins J. Cooper R. Munoz S. Cooper R. A. (2014). Rehabilitation of people with lower-limb amputations. Curr. Phys. Med. Rehabil. Rep. 2, 263–272. 10.1007/s40141-014-0068-8

[ref20] Galica A. M. Kang H. G. Priplata A. A. D’Andrea S. E. Starobinets O. V. Sorond F. A.. (2009). Subsensory vibrations to the feet reduce gait variability in elderly fallers. Gait Posture 30, 383–387. 10.1016/j.gaitpost.2009.07.005, PMID: 19632845 PMC2745077

[ref21] Gammaitoni L. Hänggi P. Jung P. Marchesoni F. (1998). Stochastic resonance. Rev. Mod. Phys. 70, 223–287. 10.1103/RevModPhys.70.223

[ref22] Geurts A. C. Mulder T. W. Nienhuis B. Mars P. Rijken R. A. (1992). Postural organization in patients with hereditary motor and sensory neuropathy. Arch. Phys. Med. Rehabil. 73, 569–572. PMID: 1622307

[ref23] Gilden D. L. (2001). Cognitive emissions of 1/f noise. Psychol. Rev. 108:33. 10.1037/0033-295x.108.1.33, PMID: 11212631

[ref24] Gilden D. L. Thornton T. Mallon M. W. (1995). 1/f noise in human cognition. Science 267, 1837–1839. 10.1126/science.7892611, PMID: 7892611

[ref25] Goldberger A. L. Amaral L. A. N. Hausdorff J. M. Ivanov P. C. Peng C. -K. Stanley H. E. (2002). Fractal dynamics in physiology: alterations with disease and aging. Proc. Natl. Acad. Sci. U. S. A. 99(Suppl. 1), 2466–2472. 10.1073/pnas.012579499, PMID: 11875196 PMC128562

[ref26] Gravelle D. C. Laughton C. A. Dhruv N. T. Katdare K. D. Niemi J. B. Lipsitz L. A.. (2002). Noise-enhanced balance control in older adults. Neuroreport 13, 1853–1856. 10.1097/00001756-200210280-00004, PMID: 12395078

[ref27] Harrison S. J. Hough M. Schmid K. Groff B. Stergiou N. (2018). When coordinating finger tapping to a variable beat the variability scaling structure of the movement and the cortical BOLD signal are both entrained to the auditory stimuli. Neuroscience 392, 203–218. 10.1016/j.neuroscience.2018.06.025, PMID: 29958941 PMC8091912

[ref28] Harrison S. J. Stergiou S. J. (2015). Complex adaptive behavior and dexterous action. Nonlinear Dynam. Psychol. Life Sci. 19, 345–394. PMID: 26375932 PMC4755319

[ref29] Hausdorff J. M. (2005). Gait variability: methods, modeling and meaning. J. Neuroeng. Rehabil. 2:19. 10.1186/1743-0003-2-19, PMID: 16033650 PMC1185560

[ref30] Hausdorff J. M. (2007). Gait dynamics, fractals and falls: finding meaning in the stride-to-stride fluctuations of human walking. Hum. Mov. Sci. 26, 555–589. 10.1016/j.humov.2007.05.003, PMID: 17618701 PMC2267927

[ref31] Hausdorff J. M. (2009). Gait dynamics in Parkinson’s disease: common and distinct behavior among stride length, gait variability, and fractal-like scaling. Chaos 19:026113. 10.1063/1.3147408, PMID: 19566273 PMC2719464

[ref32] Hausdorff J. M. Mitchell S. L. Firtion R. Peng C. K. Cudkowicz M. E. Wei J. Y.. (1997). Altered fractal dynamics of gait: reduced stride-interval correlations with aging and Huntington’s disease. J. Appl. Physiol. 82, 262–269. 10.1152/jappl.1997.82.1.262, PMID: 9029225

[ref33] Hausdorff J. M. Peng C. K. Ladin Z. Wei J. Y. Goldberger A. L. (1995). Is walking a random walk? Evidence for long-range correlations in stride interval of human gait. J. Appl. Physiol. 78, 349–358. 10.1152/jappl.1995.78.1.349, PMID: 7713836

[ref34] Hendy A. M. Lamon S. (2017). The cross-education phenomenon: brain and beyond. Front. Physiol. 8:297. 10.3389/fphys.2017.00297, PMID: 28539892 PMC5423908

[ref35] Höhne A. Ali S. Stark C. Brüggemann G. -P. (2012). Reduced plantar cutaneous sensation modifies gait dynamics, lower-limb kinematics and muscle activity during walking. Eur. J. Appl. Physiol. 112, 3829–3838. 10.1007/s00421-012-2364-2, PMID: 22391682

[ref36] Hove M. J. Suzuki K. Uchitomi H. Orimo S. Miyake Y. (2012). Interactive rhythmic auditory stimulation reinstates natural 1/f timing in gait of Parkinson’s patients. PLoS One 7:e32600. 10.1371/journal.pone.0032600, PMID: 22396783 PMC3292577

[ref37] Hunt N. McGrath D. Stergiou N. (2014). The influence of auditory-motor coupling on fractal dynamics in human gait. Sci. Rep. 4:5879. 10.1038/srep05879, PMID: 25080936 PMC4118321

[ref38] Ihlen E. A. F. Vereijken B. (2013). Identifying multiplicative interactions between temporal scales of human movement variability. Ann. Biomed. Eng. 41, 1635–1645. 10.1007/s10439-012-0724-z, PMID: 23247986

[ref39] Iliopoulos F. Nierhaus T. Villringer A. (2013). Electrical noise modulates perception of electrical pulses in humans: sensation enhancement via stochastic resonance. J. Neurophysiol. 111, 1238–1248. 10.1152/jn.00392.2013, PMID: 24353303

[ref40] Itzcovich E. Riani M. Sannita W. G. (2017). Stochastic resonance improves vision in the severely impaired. Sci. Rep. 7:12840. 10.1038/s41598-017-12906-2, PMID: 28993662 PMC5634416

[ref41] Ivanov P. C. Nunes Amaral L. A. Goldberger A. L. Havlin S. Rosenblum M. G. Stanley H. E.. (2001). From 1/f noise to multifractal cascades in heartbeat dynamics. Chaos 11, 641–652. 10.1063/1.1395631, PMID: 12779503

[ref42] Jooss A. Haberbosch L. Köhn A. Rönnefarth M. Bathe-Peters R. Kozarzewski L.. (2019). Motor task-dependent dissociated effects of transcranial random noise stimulation in a finger-tapping task versus a go/no-go task on corticospinal excitability and task performance. Front. Neurosci. 13:161. 10.3389/fnins.2019.00161, PMID: 30872997 PMC6400855

[ref43] Jordan K. Challis J. H. Newell K. M. (2007a). Speed influences on the scaling behavior of gait cycle fluctuations during treadmill running. Hum. Mov. Sci. 26, 87–102. 10.1016/j.humov.2006.10.001, PMID: 17161484

[ref44] Jordan K. Challis J. H. Newell K. M. (2007b). Walking speed influences on gait cycle variability. Gait Posture 26, 128–134. 10.1016/j.gaitpost.2006.08.010, PMID: 16982195

[ref45] Kaipust J. P. Huisinga J. M. Filipi M. Stergiou N. (2012). Gait variability measures reveal differences between multiple sclerosis patients and healthy controls. Mot. Control. 16, 229–244. 10.1123/mcj.16.2.229, PMID: 22615327

[ref46] Kaipust J. P. McGrath D. Mukherjee M. Stergiou N. (2013). Gait variability is altered in older adults when listening to auditory stimuli with differing temporal structures. Ann. Biomed. Eng. 41, 1595–1603. 10.1007/s10439-012-0654-9, PMID: 22956164

[ref47] Kello C. T. Anderson G. G. Holden J. G. Orden G. C. V. (2008). The pervasiveness of 1/f scaling in speech reflects the metastable basis of cognition. Cogn. Sci. 32, 1217–1231. 10.1080/03640210801944898, PMID: 21585450

[ref48] Kello C. T. Brown G. D. A. Ferrer-i-Cancho R. Holden J. G. Linkenkaer-Hansen K. Rhodes T.. (2010). Scaling laws in cognitive sciences. Trends Cogn. Sci. 14, 223–232. 10.1016/j.tics.2010.02.005, PMID: 20363176

[ref49] Khaodhiar L. Niemi J. B. Earnest R. Lima C. Harry J. D. Veves A. (2003). Enhancing sensation in diabetic neuropathic foot with mechanical noise. Diabetes Care 26, 3280–3283. 10.2337/diacare.26.12.3280, PMID: 14633814

[ref50] Krauss P. Tziridis K. Metzner C. Schilling A. Hoppe U. Schulze H. (2016). Stochastic resonance controlled upregulation of internal noise after hearing loss as a putative cause of tinnitus-related neuronal hyperactivity. Front. Neurosci. 10:597. 10.3389/fnins.2016.00597, PMID: 28082861 PMC5187388

[ref51] Kurita Y. Shinohara M. Ueda J. (2013). Wearable sensorimotor enhancer for fingertip based on stochastic resonance effect. IEEE Trans. Hum. Mach. Syst. 43, 333–337. 10.1109/TSMC.2013.2242886

[ref52] Legro M. W. Reiber G. D. Smith D. G. del Aguila M. Larsen J. Boone D. (1998). Prosthesis evaluation questionnaire for persons with lower limb amputations: assessing prosthesis-related quality of life. Arch. Phys. Med. Rehabil. 79, 931–938. 10.1016/S0003-9993(98)90090-9, PMID: 9710165

[ref53] Likens A. D. Amazeen P. G. Stevens R. Galloway T. Gorman J. C. (2014). Neural signatures of team coordination are revealed by multifractal analysis. Soc. Neurosci. 9, 219–234. 10.1080/17470919.2014.882861, PMID: 24517441

[ref54] Likens A. D. Fine J. M. Amazeen E. L. Amazeen P. G. (2015). Experimental control of scaling behavior: what is not fractal? Exp. Brain Res. 233, 2813–2821. 10.1007/s00221-015-4351-4, PMID: 26070902

[ref55] Lipsitz L. A. (2002). Dynamics of stabilitythe physiologic basis of functional health and frailty. J. Gerontol. A Biol. Sci. Med. Sci. 57, B115–B125. 10.1093/gerona/57.3.B115, PMID: 11867648

[ref56] Lipsitz L. A. Lough M. Niemi J. Travison T. Howlett H. Manor B. (2015). A shoe insole delivering subsensory vibratory noise improves balance and gait in healthy elderly people. Arch. Phys. Med. Rehabil. 96, 432–439. 10.1016/j.apmr.2014.10.004, PMID: 25450133 PMC4339481

[ref57] Liu W. Lipsitz L. A. Montero-Odasso M. Bean J. Kerrigan D. C. Collins J. J. (2002). Noise-enhanced vibrotactile sensitivity in older adults, patients with stroke, and patients with diabetic neuropathy. Arch. Phys. Med. Rehabil. 83, 171–176. 10.1053/apmr.2002.28025, PMID: 11833019

[ref58] Lugo E. Doti R. Faubert J. (2008). Ubiquitous crossmodal stochastic resonance in humans: auditory noise facilitates tactile, visual and proprioceptive sensations. PLoS One 3:e2860. 10.1371/journal.pone.0002860, PMID: 18682745 PMC2481403

[ref59] Magalhães F. H. Kohn A. F. (2011). Vibratory noise to the fingertip enhances balance improvement associated with light touch. Exp. Brain Res. 209, 139–151. 10.1007/s00221-010-2529-3, PMID: 21191573

[ref60] Mancini F. Bauleo A. Cole J. Lui F. Porro C. A. Haggard P.. (2014). Whole-body mapping of spatial acuity for pain and touch. Ann. Neurol. 75, 917–924. 10.1002/ana.24179, PMID: 24816757 PMC4143958

[ref61] Manor B. Costa M. D. Hu K. Newton E. Starobinets O. Kang H. G.. (2010). Physiological complexity and system adaptability: evidence from postural control dynamics of older adults. J. Appl. Physiol. 109, 1786–1791. 10.1152/japplphysiol.00390.2010, PMID: 20947715 PMC3006415

[ref62] McDonnell M. D. Abbott D. (2009). What is stochastic resonance? Definitions, misconceptions, debates, and its relevance to biology. PLoS Comput. Biol. 5:e1000348. 10.1371/journal.pcbi.1000348, PMID: 19562010 PMC2660436

[ref63] McKeon P. O. Booi M. J. Branam B. Johnson D. L. Mattacola C. G. (2010). Lateral ankle ligament anesthesia significantly alters single limb postural control. Gait Posture 32, 374–377. 10.1016/j.gaitpost.2010.06.016, PMID: 20663671

[ref64] Meyer P. F. Oddsson L. I. E. De Luca C. J. (2004). Reduced plantar sensitivity alters postural responses to lateral perturbations of balance. Exp. Brain Res. 157, 526–536. 10.1007/s00221-004-1868-3, PMID: 15029466

[ref65] Mildren R. L. Strzalkowski N. D. J. Bent L. R. (2016). Foot sole skin vibration perceptual thresholds are elevated in a standing posture compared to sitting. Gait Posture 43, 87–92. 10.1016/j.gaitpost.2015.10.027, PMID: 26669957

[ref66] Moss F. Ward L. M. Sannita W. G. (2004). Stochastic resonance and sensory information processing: a tutorial and review of application. Clin. Neurophysiol. 115, 267–281. 10.1016/j.clinph.2003.09.014, PMID: 14744566

[ref67] Murphy E. F. (1984). Sockets, linings, and interfaces. Clin. Prosthet. Orthot. 8, 4–10.

[ref68] Nobusako S. Osumi M. Matsuo A. Fukuchi T. Nakai A. Zama T.. (2018). Stochastic resonance improves visuomotor temporal integration in healthy young adults. PLoS One 13:e0209382. 10.1371/journal.pone.0209382, PMID: 30550570 PMC6294379

[ref69] Nozaki D. Collins J. J. Yamamoto Y. (1999a). Mechanism of stochastic resonance enhancement in neuronal models driven by 1/f noise. Phys. Rev. E Stat. Phys. Plasmas Fluids Relat. Interdiscip. Topics 60, 4637–4744. 10.1103/physreve.60.4637, PMID: 11970325

[ref70] Nozaki D. Mar D. J. Grigg P. Collins J. J. (1999b). Effects of colored noise on stochastic resonance in sensory neurons. Phys. Rev. Lett. 82, 2402–2405. 10.1103/PhysRevLett.82.2402

[ref71] Nozaki D. Yamamoto Y. (1998). Enhancement of stochastic resonance in a FitzHugh-Nagumo neuronal model driven by colored noise. Phys. Lett. A 243, 281–287. 10.1016/S0375-9601(98)00247-3

[ref72] Pacchierotti C. Sinclair S. Solazzi M. Frisoli A. Hayward V. Prattichizzo D. (2017). Wearable haptic systems for the fingertip and the hand: taxonomy, review, and perspectives. IEEE Trans. Haptics 10, 580–600. 10.1109/TOH.2017.2689006, PMID: 28500008

[ref73] Phinyomark A. Larracy R. Scheme E. (2020). Fractal analysis of human gait variability via stride interval time series. Front. Physiol. 11:333. 10.3389/fphys.2020.00333, PMID: 32351405 PMC7174763

[ref74] Priplata A. A. Niemi J. B. Harry J. D. Lipsitz L. A. Collins J. J. (2003). Vibrating insoles and balance control in elderly people. Lancet 362, 1123–1124. 10.1016/S0140-6736(03)14470-4, PMID: 14550702

[ref75] Priplata A. A. Patritti B. L. Niemi J. B. Hughes R. Gravelle D. C. Lipsitz L. A.. (2006). Noise-enhanced balance control in patients with diabetes and patients with stroke. Ann. Neurol. 59, 4–12. 10.1002/ana.20670, PMID: 16287079

[ref76] Qu G. Fan B. Fu X. Yu Y. (2019). The impact of frequency scale on the response sensitivity and reliability of cortical neurons to 1/fβ input signals. Front. Cell. Neurosci. 13:311. 10.3389/fncel.2019.00311, PMID: 31354432 PMC6637762

[ref77] Quai T. M. Brauer S. G. Nitz J. C. (2005). Somatosensation, circulation and stance balance in elderly dysvascular transtibial amputees. Clin. Rehabil. 19, 668–676. 10.1191/0269215505cr857oa, PMID: 16180604

[ref78] Raudenbush S. W. Bryk A. S. (2002). Hierarchical linear models: Applications and data analysis methods. 2nd Edn. Thousand Oaks, CA, USA: Sage Publications.

[ref79] Richardson K. A. Imhoff T. T. Grigg P. Collins J. J. (1998). Using electrical noise to enhance the ability of humans to detect subthreshold mechanical cutaneous stimuli. Chaos 8, 599–603. 10.1063/1.166341, PMID: 12779763

[ref80] Sasaki H. Todorokihara M. Ishida T. Miyachi J. Kitamura T. Aoki R. (2006). Effect of noise on the contrast detection threshold in visual perception. Neurosci. Lett. 408, 94–97. 10.1016/j.neulet.2006.08.054, PMID: 16996210

[ref81] Sejdić E. Fu Y. Pak A. Fairley J. A. Chau T. (2012). The effects of rhythmic sensory cues on the temporal dynamics of human gait. PLoS One 7:e43104. 10.1371/journal.pone.0043104, PMID: 22927946 PMC3424126

[ref82] Sinha R. van den Heuvel W. J. Arokiasamy P. (2011). Factors affecting quality of life in lower limb amputees. Prosthetics Orthot. Int. 35, 90–96. 10.1177/0309364610397087, PMID: 21515894

[ref83] Stephen D. G. Wilcox B. Niemi J. B. Franz J. Kerrigan D. C. D’Andrea S. E. (2012). Baseline-dependent effect of noise-enhanced insoles on gait variability in healthy elderly walkers. Gait Posture 36, 537–540. 10.1016/j.gaitpost.2012.05.014, PMID: 22739049 PMC3978195

[ref84] Stergiou N. Harbourne R. T. Cavanaugh J. T. (2006). Optimal movement variability: a new theoretical perspective for neurologic physical therapy. J. Neurol. Phys. Ther. 30:120. 10.1097/01.NPT.0000281949.48193.d9, PMID: 17029655

[ref85] Stergiou N. Kent J. A. McGrath D. (2016). Human movement variability and aging. Kinesiol. Rev. 5, 15–22. 10.1123/kr.2015-0048

[ref86] Stroe-Kunold E. Stadnytska T. Werner J. Braun S. (2009). Estimating long-range dependence in time series: an evaluation of estimators implemented in R. Behav. Res. Methods 41, 909–923. 10.3758/BRM.41.3.909, PMID: 19587208

[ref87] Sueda Y. Hattori M. Sawada H. Egi H. Ohdan H. Ueda J. (2013). “Improvement of tactile sensitivity by stochastic resonance effect—Applications to surgical grasping forceps” in *35th Annual International Conference of the IEEE Engineering in Medicine and Biology Society (EMBC);* July 2013; 4601–4604. 10.1109/EMBC.2013.6610572 24110759

[ref88] Toosizadeh N. Mohler J. Marlinski V. (2018). Low intensity vibration of ankle muscles improves balance in elderly persons at high risk of falling. PLoS One 13:e0194720. 10.1371/journal.pone.0194720, PMID: 29579098 PMC5868830

[ref89] Van Orden G. C. Holden J. G. Turvey M. T. (2003). Self-organization of cognitive performance. J. Exp. Psychol. Gen. 132:331. 10.1037/0096-3445.132.3.331, PMID: 13678372

[ref90] Van Orden G. C. Kloos H. Wallot S. (2011). “Living in the pink: intentionality, wellbeing, and complexity” in Philosophy of complex systems. ed. Hooker C. (North Holland: Elsevier), 629–672.

[ref91] Vaz J. R. Groff B. R. Rowen D. A. Knarr B. A. Stergiou N. (2019). Synchronization dynamics modulates stride-to-stride fluctuations when walking to an invariant but not to a fractal-like stimulus. Neurosci. Lett. 704, 28–35. 10.1016/j.neulet.2019.03.040, PMID: 30922850 PMC7196175

[ref92] Voytek B. Kramer M. A. Case J. Lepage K. Q. Tempesta Z. R. Knight R. T.. (2015). Age-related changes in 1/f neural electrophysiological noise. J. Neurosci. 35, 13257–13265. 10.1523/JNEUROSCI.2332-14.2015, PMID: 26400953 PMC4579381

[ref93] Wan A. H. Wong D. W. Ma C. Z. Zhang M. Lee W. C. (2016). Wearable vibrotactile biofeedback device allowing identification of different floor conditions for lower-limb amputees. Arch. Phys. Med. Rehabil. 97, 1210–1213. 10.1016/j.apmr.2015.12.016, PMID: 26763948

[ref94] Wells C. Ward L. M. Chua R. Inglis J. T. (2005). Touch noise increases vibrotactile sensitivity in old and young. Psychol. Sci. 16, 313–320. 10.1111/j.0956-7976.2005.01533.x, PMID: 15828979

[ref95] West B. J. Latka M. (2005). Fractional Langevin model of gait variability. J. Neuroeng. Rehabil. 2, 1–9. 10.1186/1743-0003-2-24, PMID: 16076394 PMC1224863

[ref96] Winter D. A. (2009). Biomechanics and motor control of human movement. 4th Edn. Waterloo, Ontario, Canada: Wiley.

[ref97] Wurdeman S. R. Stevens P. M. Campbell J. H. (2018). Mobility Analysis of AmpuTees (MAAT I): quality of life and satisfaction are strongly related to mobility for patients with a lower limb prosthesis. Prosthetics Orthot. Int. 42, 498–503. 10.1177/0309364617736089, PMID: 28990467 PMC6146310

[ref98] Xue Y. Rodriguez S. Bogdan P. (2016). “A spatio-temporal fractal model for a CPS approach to brain-machine-body interfaces” in *Design, Automation and Test in Europe Conference and Exhibition (DATE);* March, 2016; 642–647.

[ref99] Zeni J. A. Jr. Richards J. G. Higginson J. S. (2008). Two simple methods for determining gait events during treadmill and overground walking using kinematic data. Gait Posture 27, 710–714. 10.1016/j.gaitpost.2007.07.007, PMID: 17723303 PMC2384115

[ref100] Zwaferink J. B. J. Hijmans J. M. Schrijver C. M. Schrijver L. K. Postema K. van Netten J. J. (2018). Mechanical noise improves the vibration perception threshold of the foot in people with diabetic neuropathy. J. Diabetes Sci. Technol. 14, 16–21. 10.1177/1932296818804552, PMID: 30328708 PMC7189161

